# The implications of the disease model and psychological model on eating disorder treatment

**DOI:** 10.1007/s40519-023-01527-6

**Published:** 2023-02-11

**Authors:** Riccardo Dalle Grave

**Affiliations:** Department of Eating and Weight Disorders, Villa Garda Hospital, Via Monte Baldo, 89, 37016 Garda, VR Italy

## Introduction

The egosyntonic nature of some expressions of eating-disorder psychopathology, such as the adoption of extreme weight control behaviours, the active maintenance of low weight, and the preoccupation with shape, weight and eating control, has been identified by several research teams as the main reason behind the reluctance of the people with eating disorders to seek, engage in and persevere with treatment [1]. However, understanding why a person is ambivalent toward the treatment and continues to adopt extreme weight control behaviours and maintain a condition of low weight, despite the adverse effects on their physical health, psychological well-being and interpersonal relationships, is a widely debated issue.

Two main theoretical models have been proposed to explain the development and maintenance of eating-disorder psychopathology, and the egosyntonic nature of some of its expressions, namely (1) the disease model (also called the medical model) and (2) the psychological model.

This editorial focuses on the clinical implications of the two models. The different conceptualizations of eating-disorder psychopathology, the nature of the involvement of patients, significant others, and clinicians, the general treatment strategies, and the strengths and weaknesses of the two models are discussed. Finally, based on the above, several clinical considerations are made.

## The disease model

The disease model postulates that the features of eating disorders, including strict dieting, other extreme weight control behaviours, binge-eating and purging, fear of gaining weight, and preoccupation with shape and weight, are the symptoms of a specific "disease", namely anorexia nervosa, bulimia nervosa, binge-eating disorder or other eating disorders [2]. The model is rooted in the medical model of mental disorders and considers eating disorders in much the same way as physical diseases [3].

The disease model has important implications for the treatment of eating disorders. If the eating disorder is a disease, the onus is on the clinicians to defeat it by adopting an active and prescriptive approach. Patients, on the other hand, are not considered to be in control of their behaviour—the disease model posits that the eating disorder controls them. Under this model, patients must, therefore, play an uncritical role in treatment. Indeed, even when it asks the patient to adopt an active role, it is a "mindless" model, i.e., a model that does not help the patients to understand their illness because it only tries to explain it. Patients are often asked not to trust their thoughts about shape, weight and eating, as these are considered symptoms of their disease generated by the “ill” part of their brain. To get well, they must follow the instructions of their therapists. To ensure treatment success, their parents or significant others are often actively involved as “controllers”, overseeing that the patient follows the therapeutic prescriptions.

The disease model is widely adhered to by individual providers of medical, psychiatric and nutritional treatment of eating disorders and in many clinical services for eating disorders. The main advantage of the disease model is that it does not blame the patient or parents for the development of the eating disorder, as this is viewed as an external agent, akin to any other pathogen. Another advantage is to favour the allocation of economic resources to the research and treatment of eating disorders as it would be done for heart problems and other serious diseases.

However, this model also has some flaws. First and foremost, the purported biological causes of eating disorders have not yet been identified. Although the biological mechanisms underlying eating disorders are the subject of active research [4], and some progress has been made in understanding neurobiology and genetic predisposition in anorexia nervosa [5, 6], there are no biomarkers of eating disorders to be detected through blood tests or brain scans. This means that there is no possibility of acquiring independent, objective data in support of the DSM-5 diagnosis of these “diseases”. Moreover, there have been as yet no satisfactory results from biological and pharmacological treatments for eating disorders [7].

Second, implementing a disease model using an external control regime, as it occurs in some impatient and intensive specialized eating disorders units, can have two faces. The first is good applicability and short-term positive outcome in compliant patients during the intensive treatment, which often translates into a second phase after discharge at home where the "uncontrolled" patient often relapses [8].

Third, conceptualizing their disorder as a disease robs the afflicted of the opportunity to understand the psychological functions—perceived and actual—associated with the control of shape, weight and eating. It fails to take into account the individual's experience, or other "external" factors which may be influencing their behaviour. It reduces the human brain to a complex biological structure, rather than the exquisitely sophisticated learning engine that it is.

Finally, even if the idea that eating disorders are diseases like any other has been seen as a powerful way of reducing stigma and discrimination, a strong emphasis on their biological origin may paradoxically yield the opposite outcome, because they are considered a heritable and “immutable” part of the person. Indeed, it implies a dichotomy between “normal” and “abnormal”, and between mentally “healthy” or “ill”. This contrasts with the view of eating disorders as a continuum of mental experience, and an extension of the dieting patterns and body dissatisfaction experienced by many people of Western cultures [9] when these features become so extreme to determine a clinically significant impairment.

## The psychological model

Psychological models are based on hypothetical constructs [10]. They postulate that people have an eating disorder as a result of the way they perceive, process, or make sense of different life experiences, which may be dependent on a wide range of factors, including, among others, learning, personality, stress, cognition, self-efficacy, and early life experiences. Several psychological theories have been proposed to explain the egosyntonic nature of eating disorders, and account for their onset and persistence [11–15]. Here, I focus only on the theoretical model behind enhanced cognitive behaviour therapy (CBT-E) [15, 16], because it is the one that I know best and has had an important influence on the evidence-based treatment of eating disorders [17]. That being said, some of the considerations in the following paragraphs can also be applied to other treatments based on the psychological model.

CBT-E addresses the patients' individual psychopathology, not the DSM-5 diagnosis. It never uses "prescriptive" procedures, and never asks a patient to do anything that they are not willing to. As such, the goals of the first phase of the treatment, which lasts about four weeks, do not include weight regain, but are instead education and engagement. Early sessions aim to help the person understand the psychological nature of their eating disorder, including the perceived positive but actually dysfunctional consequences of basing their self-worth on their control of shape, weight and eating, thereby breaking down their resistance to change. At the end of this phase, the person is asked to make the decision to change. If they do not agree to make the change, CBT-E cannot be continued, and another treatment, which does not require their active role (e.g., family based treatment in adolescents or hospitalization to stabilize the medical and psychiatric conditions), should be considered, but in my experience this seldom occurs. In the second, “active” phase of treatment, the patient learns to address their individual eating disorder psychopathology, and to tackle weight gain (if indicated), via a flexible and personalized set of sequential cognitive and behavioural strategies and progressive education. To achieve cognitive change, patients are encouraged to observe, using real-time self-monitoring, how their cognitive and behavioural processes operate in real life. Armed with this information, they are then asked to make gradual behavioural changes and to analyse the effects and implications of these on their thinking. In the later stages of CBT-E, the treatment focuses on helping patients recognize the early warning signs of eating-disorder mindset reactivation, and teaching them how to decentre from it quickly, thereby preventing relapse.

The main advantages of the psychological model, as adopted by CBT-E, are that it helps patients to understand that dysfunctional self-evaluation, i.e., that predominantly based on shape, weight and eating control, is detrimental in the long-term. They learn that there are ways, other than weight control, of developing a functional self-evaluation system that is not deleterious to their physical and mental health. This exploration increases their understanding and facilitates their empowerment and the development of a collaborative therapeutic relationship between patient and clinician. Furthermore, even if the patient decides to discontinue treatment, they have generally improved their knowledge of the psychological function of their eating disorder in a stress-free context, thereby leaving them more open to treatment at a future time.

In addition, the historical analysis of life events carried out in the later phases of the CBT-E, when patients have developed a more functional self-evaluation schema, can help them to normalize their experience, and distance themselves from the eating-disorder mindset. It allows them to dispel any blame associated with their illness, without having to adopt a disease model explanation.

The efficacy and effectiveness of CBT-E have been demonstrated by several studies [18]. However, it is acknowledged that a treatment based on the psychological model is not suitable for all patients. It is dependent on the active engagement of patients in overcoming their own eating disorder, which is not always possible to obtain. It may also prove ineffective in patients who are actively engaged, which may increase their sense of helplessness towards the eating disorder and delay the implementation of other treatments. As with other treatments, including the evidence-free eclectic or integrative psychological approaches commonly offered in routine clinical practice [19], there is a risk of a patient’s clinical conditions deteriorating during this time.

## Clinical considerations

The disease model and the psychological model, as described above, present marked differences, including the conceptualization of the eating disorder, the role of the therapists, the patients, and the involvement of significant others. Unlike the disease model, the psychological model does not separate the illness from the patient (externalization). The patients are active participants in their own recovery, while parents and significant others are involved as “helpers” rather than “controllers”. The therapist does not prescribe “a cure”, but instead works together with their patient to understand and address the psychological and behavioural mechanisms that are maintaining their individual eating-disorder presentation (collaborative empiricism).

The two models also use different vocabulary to describe eating-disorder psychopathology, which can itself be a source of confusion. Although in everyday English, but also in many non-English languages, the terms “illness” and “disease” are used interchangeably, in scientific English parlance, disease is the underlying biological pathology, while illness is a term that should be used to describe the personal experience of symptoms and suffering [20].

Under the disease model, patients are taught to refer to the eating disorder as a "voice" produced by biological alteration inside their mind, which orders them to restrict food, purge, exercise or engage in the other behaviours typical of an eating disorder. Patients are encouraged to say things to themselves like, ‘That is not you speaking; that is your disease,’ or ‘You are dieting and exercising excessively because you have an eating disorder". These comments contradict the psychological treatment models, as they imply that an external force is controlling the patients and that they do not have the option to regain control of themselves. In contrast, psychological treatments like CBT-E posit that externalizing the eating disorder in this way can perpetuate a passive stance, in which the patients may continue practicing harmful behaviours in the belief that they are powerless to take matters into their own hands.

Many researchers and clinicians adhering to the disease model utilize a biopsychosocial, rather than purely biological, conceptualization [21] of the underlying causes and maintenance factors of eating disorders. However, this rather nebulous approach encourages undisciplined eclecticism and therapeutic drift, especially in multidisciplinary clinical services for eating disorders. These can rely on a mishmash of medical, psychological, and social intervention procedures stemming from different and conflicting theories. For example, the psychologist may swear by psychoanalytical theory, and the dietician may adopt some basic cognitive behavioural procedures and strategies, while psychiatrists and physicians are likely to follow a prescriptive biomedical model. In other words, each member of the team follows the theory and practice of their profession, pursuing field-specific therapeutic goals. Although each of these approaches undoubtedly has its merits and will benefit the patient to a certain degree, their coadministration inevitably entails some significant drawbacks. Though ostensibly playing the same game, without overarching orchestration, the team is unlikely to win—they may all individually be outstanding players, but a shared game plan will be necessary for success. In such scenarios, patients receive conflicting information on how they should experience and address their eating disorders, causing confusion and a sense of powerlessness in people who are already unstable. This disempowerment may be enough to compromise the effectiveness that the individual treatments may otherwise have had.

From a research perspective, without a unifying theory, it is also almost impossible to understand which parts of treatment do and do not work, and in whom, creating an insurmountable barrier to any meaningful evolution in treatment approaches.

Given the above and the current lack of demonstrated efficacy of treatments based on biological interventions, I urge clinicians and clinical services to design their care pathways around a unified theoretical model coherent with the evidence-based psychological interventions available to date. Even when there is a clinical need to implement specific medical interventions in association with psychological treatment, it is advisable to maintain a shared language and uniform multidisciplinary approach based on the theory and the psychological treatment on which the patient is treated. This will help prevent confusion and discontinuity of care among patients, empowering them to take control of their own thoughts and behaviours, and thereby drive their own recovery.

## Data Availability

Not applicable.
